# Differential Expression Profile of *ZFX* Variants Discriminates Breast Cancer Subtypes

**DOI:** 10.29252/.23.1.47

**Published:** 2019-01

**Authors:** Fatemeh Pourkeramati, Malek Hossein Asadi, Shahryar Shakeri, Alireza Farsinejad

**Affiliations:** 1 Department of Biotechnology, Institute of Science and High Technology and Environmental Sciences, Graduate University of Advanced Technology, Kerman, Iran; 2 Pathology and Stem Cell Research Center, Kerman University of Medical Sciences, Kerman, Iran

**Keywords:** Breast neoplasms, Neoplastic stem cells, RNA splicing

## Abstract

**Background:**

ZFX is a transcriptional regulator in embryonic stem cells and plays an important role in pluripotency and self-renewal. *ZFX* is widely expressed in pluripotent stem cells and is down-regulated during differentiation of embryonic stem cells. *ZFX* has five different variants that encode three different protein isoforms. While several reports have determined the overexpression of *ZFX* in a variety of somatic cancers, the expression of *ZFX*-spliced variants in cancer cells is not well-understood.

**Methods:**

We investigated the expression of *ZFX* variants in a series of breast cancer tissues and cell lines using quantitative PCR.

**Results:**

The expression of *ZFX* variant 1/3 was higher in tumor tissue compared to marginal tissue. In contrast, the *ZFX* variant 5 was down-regulated in tumor tissues. While the *ZFX* variant 1/3 and *ZFX* variant 5 expression significantly increased in low-grade tumors, *ZFX* variant 4 was strongly expressed in high-grade tumors, demonstrating lymphatic invasion. In addition, our result revealed a significant association between the HER2 status and the expression of *ZFX*-spliced variants.

**Conclusion:**

Our data suggest that the expression of *ZFX*-spliced transcripts varies between different types of breast cancer and may contribute to their tumorigenesis process. Hence, *ZFX*-spliced transcripts could be considered as novel tumor markers with a probable value in diagnosis, prognosis, and therapy of breast cancer.

## INTRODUCTION

Cancer stem cells (CSCs) are a sub-population of tumor cells with stem cell properties, including self-renewal and pluripotency. CSCs play important roles in the immunological and genetic heterogeneity of tumors, tumor initiation, metastasis, and resistance to therapy[1,2]. In recent years, CSCs have been shown to express stemness factors such as *OCT4*, *SOX2*, *NANOG* and *KLF4* in somatic cancers and attention is being focused on the development of novel drugs and treatment procedures, specifically targeting CSCs[3,4]. The *ZFX* (zinc finger protein X-linked) gene encodes a member of the kruppel C_2_H_2_-type zinc-finger protein family that is located on the X chromosome (at Xp22.12) and is structurally similar to a related gene on the Y chromosome (*ZFY*)[5]. *ZFX* is widely expressed in pluripotent stem cells and is down-regulated during differentiation of embryonic stem cells[6,7]. ZFX is highly conserved among vertebrates and contains three different domains: an acidic transcriptional activation domain, a nuclear localization sequence, and a DNA-binding domain consisting of 13 C_2_H_2_-type zinc-fingers. *ZFX* has five different variants that encode three different protein isoforms. *ZFX* variants 1 and 3 (isoform I) have been found to be overexpressed in the diffused type of gastric cancer as well as different tumor grades[8], whereas the *ZFX* variant 5 transcript (isoform III) is heterogeneous in gastric specimens and has a positive correlation with tumor size[9].

Breast cancer is the most frequently diagnosed cancer and the leading cause of cancer deaths among women worldwide, with an estimated 1.7 million new cases and 521,900 deaths in 2012. Breast cancer alone accounts for 25% of all cancer cases and 15% of all cancer deaths among women[10]. Although breast cancer is often thought of as a single disease, increasing evidence suggests that there are multiple subtypes of breast cancer that show different rates of occurrence in different groups. Based on molecular markers, breast cancer is classified into four subtypes as triple-negative, HER2 over-expressing, luminal A, and luminal B. The triple-negative type of breast cancer indicates the lack of expression of estrogen receptors (ERs), *progesterone* receptors (PR), and human epidermal growth factor receptor 2 (HER2)[11]. Although the triple-negative subtype makes up only about 15% of breast cancer diagnoses, it is often aggressive and unresponsive to hormone therapy. HER2 over-expression tumors have extra copies of the *HER2* gene, leading to the up-regulation of the growth-enhancing proteins. Luminal A and B subtypes are ER-positive (ER+). Luminal A tumors have a prevalence of 30-70% and grow very slowly, whereas luminal B tumors have a prevalence of 10-20% and grow more quickly[12,13].

Recently, *ZFX* has been found to be overexpressed in different cancer types[14-19]. Inhibition of *ZFX* expression in different cancer cells by RNAi resulted in significantly impaired cell proliferation, increased apoptosis, and arrest in the G1 phase of the cell cycle[20-23]. While several reports have determined the expression of *ZFX* in tumors and stem cells, there is little information about the discriminating expression of *ZFX* splice variants in cancer. In this study, we investigated the potential expression of different variants of *ZFX* in human breast tumors and a series of stem and cancer cell lines to evaluate the variants manner.

## MATERIALS AND METHODS

### Clinical sample collection

Prior to patients’ participation, the Iran National Tumor Bank obtained the participants’ written informed consent. The breast tumor and non-tumoral specimens were then obtained from the Iran National Tumor Bank, founded by the Cancer Institute of Tehran University of Medical Sciences (Tehran, Iran). Surgical biopsy specimens from 40 female patients with ductal and seven female patients with lobular breast cancer were snap-frozen in liquid nitrogen and stored at -185 °C until being used for RNA extraction. The records of clinicopathological parameters for each sample were also obtained. The Ethics Committee of the Kerman Graduate University of Advanced Technology (Kerman, Iran) approved the experiment procedure.

### Cell lines and cell culture

MCF7, SK-BR-3, *and* NCCIT (*pluripotent* embryonic carcinoma; *teratocarcinoma*), cell lines were maintained in RPMI 1640 medium (Gibco, USA) supplemented with penicillin/streptomycin (100 U/ml and 100 mg/ml, respectively) and 10% fetal bovine serum in a humidified atmosphere of 5% CO_2_ incubator at 37 °C. MDA-MB-231 (human breast cancer) were cultured in Dulbecco’s modified Eagle medium (DMEM) containing 10% FBS. Passaging was routinely performed with 0.25% Trypsin/EDTA.

### Primer design for different variants of *ZFX*

**Fig. 1:**
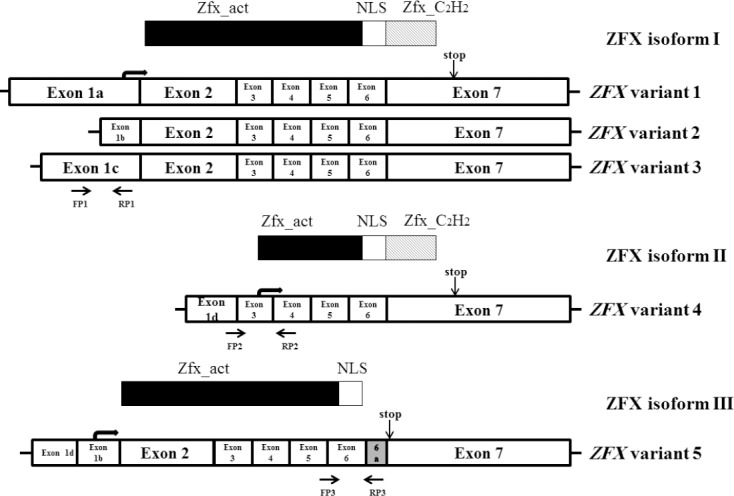
Schematic diagram of the exon structure and protein domains of *ZFX* variants. Arrows show PCR primer positions. Zfx_act, transcriptional activation domain of ZFX; Zfx_C_2_H_2_, zinc finger domain; NLS, nuclear localization signal

In humans, the *ZFX* gene can potentially encode five different variants, and three distinct protein isoforms (Fig. 1). Three variants (variants 1, 2, and 3) of *ZFX* have the same coding region and different 5’UTR regions and so encode the same protein, isoform I. *ZFX* variant 4 lacks exon 2 and encodes isoform II, and *ZFX* variant 5 is just like the *ZFX* variant 1 plus a novel exon, flanked by exons 6 and 7, which encode ZFX isoform III. Specific primers were designed for *ZFX* variants (*ZFX* variants 1/2/3*, ZFX* variant 4, and *ZFX* variant 5) and *β-actin* (as an internal control) mRNAs using Gene Runner software version 5.0.47 beta (Table 1). The expression of these variants were analyzed by quantitative PCR in tumoral and non-tumoral tissues of the breast. Electrophoresis of the PCR products on agarose gel demonstrated a single band with the expected size for the *ZFX* variants and *β-actin* transcript. Then the correlations between the expression of *ZFX* variants with the tumor’s clinicopathological properties such as grades, stages, subtypes, tumor size, age, and the status of HER2, P53, ER, and PR factors in breast cancer tissues were investigated (Table 2).

**Table 1:** Designed primers for *ZFX* variants and *β-actin*

Name	GenBank accession number	Sequence	Product Length (bp)
*ZFX* variant 1/3	NM_003410.3 NM_001178084.1 NM_001178085.1	FP1: TTCTTGCTATATTGCCCCAGG	129
		RP1: ACAGCTCAGGGAACAGACG	
*ZFX* variant 4	NM_001178086.1	FP2: CGTTCGTCCGTAGATGATGC	193
		RP2: CAGGCTCACTCTCCACAATG	
*ZFX* variant 5	NM_001178095.1	FP3: GGCAGCAGCTTATGGTAATAATTC	177
		RP3: CATGGAACTCGTGCGCCCTCA	
*β-actin*	NM_001101.3	F: ACCACCTTCAACTCCATCATG	123
		R: CTCCTTCTGCATCCTGTCG	

**Table 2:** The association between the expression of *ZFX* variants and clinicopathological parameters of breast cancer tissues

Characteristics	Numbers (%)	*p* value		

		*ZFX* variant 1/3	*ZFX* variant 4	*ZFX* variant 5
Age				
<45 years	21 (44.7)	0.074	0.78	0.89
≥45 years	26 (55.4)			
Tumor size				
<4 cm	20 (42.5)	0.81	0.64	0.018*
≥4 cm	27 (57.5)			
Tumor types				
Ductal carcinoma	40 (85.1)	0.42	0.043*	0.61
Lobular carcinoma	7 (14.9)			
Tumor grades				
Low	13 (27.7)	0.048*	0.034*	0.01**
High	27(67.5)			
ER status				
Negative	9 (22)	0.372	0.037 *	0.035*
Positive	32 (78)			
PR status				
Negative	16 (39)	0.039*	0.046*	0.583
Positive	25 (61)			
HER2 status				
Negative	32 (76.2)	0.025*	0.022*	0.036*
Positive	10 (23.8)			
P53 status				
Negative	20 (55.6)	0.277	0.639	0.046*
Positive	16 (44.4)			
Stage				
Low	30(63.8)17	0.026*	0.048*	0.481
High	(36.2)			
Subtype				
Luminal A	20 (42.6)	0.025*	0.23	0.83
Luminal B	5 (10.6)			
Triple negative	6 (12.8)			
HER2 Type	3 (6.4)			
Not classified	13 (27.6)			
Lymphatic invasion				
Negative	19 (42.2)	0.022*	0.05*	0.71
Positive	26 (57.8)			
Necrosis present				
Negative	26 (62)	0.39	0.13	0.05*
Positive	16 (48)			

* *p* < 0.05;

** *p* < 0.01

### RNA extraction, cDNA synthesis, and real-time PCR

The total RNA was extracted from the frozen tissue specimens and cancer cells, using Trizol solution (Invitrogen, USA), according to the manufacturer’s instructions. The quality of the extracted RNA was examined by UV spectrophotometry (260/280-nm ratio) as well as by the visual observation of samples on 1% agarose gel electrophoresis. To eliminate the genomic DNA, the RNase-free DNase (Fermentas, Lithuania) treatment of total RNA was performed, as per the manufacturer’s protocol. The first strand of cDNA was synthesized by using 1 µg RNA, 200 U/µl MMLV reverse transcriptase (Fermentas, Lithuania), 20U RNase inhibitor, dNTP mix (final concentration of 1 mM) with random hexamer priming in a 20-µl reaction. For each sample, a no reverse transcriptase control was simultaneously used to detect any potential contamination with genomic DNA.

Quantitative real-time RT-PCR was performed using SYBR Premix Ex Taq™ II (Takara, Japan) on ABI Step One Plus real-time quantitative PCR system (Life Technologies, USA). A PCR reaction mixture was prepared (as per manufacturer’s instructions), and PCR was performed with the following thermal cycles: initiation at 95 °C for 30 seconds, amplification cycles for 45 cycles with denaturation at 95 °C for 5 seconds, and annealing and extending at 60 °C for 30 seconds (adjusted according to primer’s Tm). PCR products were visualized on 1.5% agarose gel with ethidium bromide staining. A dilution series of cDNA concentrations were used to establish a standard curve for assessing the reaction efficiency. RT-PCR results were normalized by the reference gene *β-actin*. The relative expression of each gene was calculated by the 2^-∆∆Ct^ method.

### Differentiation of NCCIT cell lines by *trans* retinoic acid treatment

Differentiation of NCCIT cells was induced as described earlier[24]. Briefly, cells were seeded at a density of 2 × 10^6^ cells in DMEM/F12 medium (Invitrogen, USA) containing 10% fetal bovine serum and penicillin/streptomycin (100 U/ml and 100 mg/ml, respectively) and treated with 10 μM *trans* retinoic acid (Sigma-Aldrich, St. Louis, MO, USA) every two days for up to 14 days. Then the cells were harvested at different time points and used for RNA extraction. Differential biomarkers were assessed to verify the differentiation procedure.

### Statistical analysis

Experiments were replicated three times, and the normality of value distribution was tested by the Kolmogorov-Smirnov test. The differences in gene expression between the two groups were analyzed using an unpaired *t*-test and one-way ANOVA, performed by SPSS 16.0 and REST 2009 software. A *p* value less than 0.05 was considered statistically significant. The Kruskal-Wallis and Mann-Whitney nonparametric tests were used to determine the difference between the groups with a low sample size.

## RESULTS

### Expression profile of *ZFX* variants in breast cancer cell lines

**Fig. 2:**
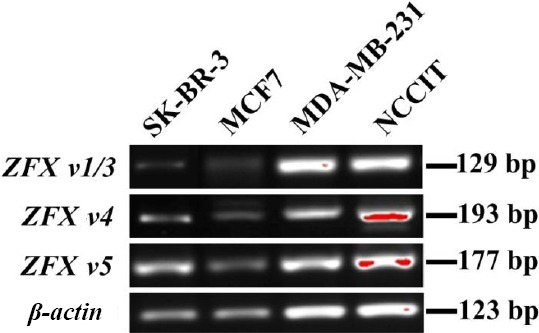
Expression pattern of *ZFX* variants in NCCIT and breast cancer cell lines in comparison to *β*-actin.

The expression of *ZFX* variants were evaluated in the breast cancer cell lines; MCF7, SK-BR-3, MDA-MB-231, and pluripotent embryonic carcinoma; NCCIT. These *ZFX* variants were expressed in embryonic stem cells (NCCIT) as well as in breast cancer cell lines (Fig. 2).

### Overexpression of *ZFX* variant 1/3 in low-grade ductal breast tumors

**Fig. 3:**
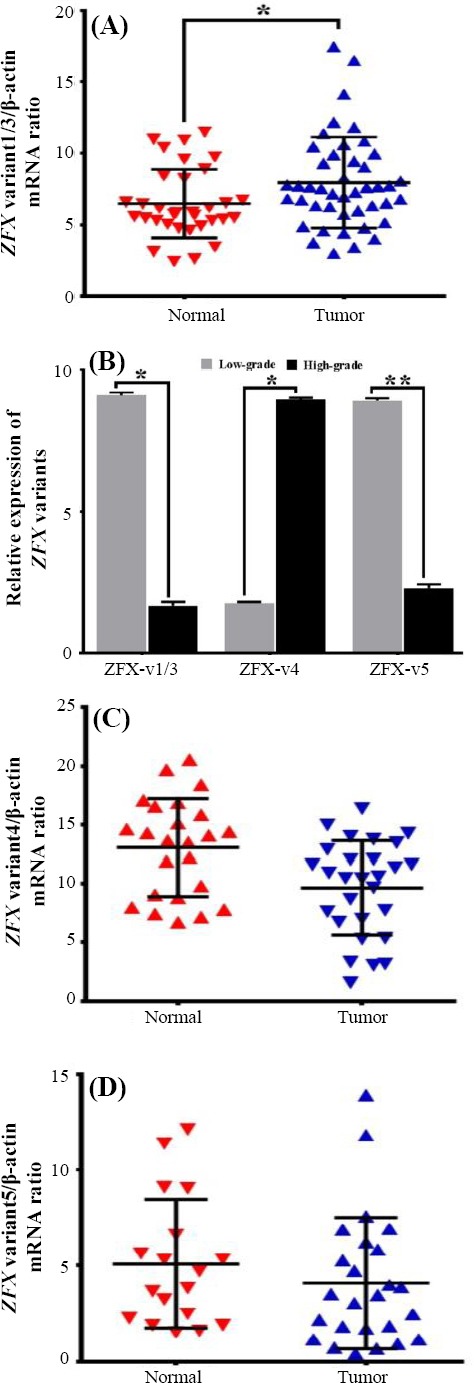
Histograms comparing the results obtained by quantitative PCR. The relative expression of *ZFX* variant 1/3 (A), variant 4 (C), and variant 5 (D) to *β*-actin in tumors vs. non-tumor tissues. The relative expression of *ZFX*-spliced variants in high-grade vs. low-grade tumors (B).

**Fig. 4:**
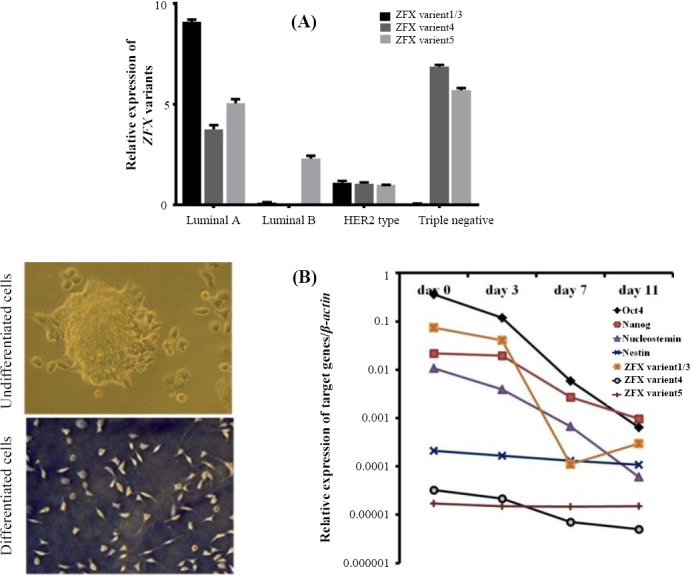
Histograms showing the relative expression of *ZFX*-spliced variant expression in different breast cancer subtypes (A). Schematic curves comparing the expressions of *OCT4*, *NANOG*, *Nucleostemin*, *Nestin*, and *ZFX* variants in undifferetiated and diferentiated NCCIT cells (B).

Our results revealed that the expression of *ZFX* variant 1/3 was higher in tumor tissues compared to non-tumor apparent tissues of the breast (*p* < 0.05; Fig. 3A). As shown in Table 2, the *ZFX* variant 1/3 expression level was higher in low-grade (grades II and I) ductal tumors compared to high-grade (grade III) ones (*p* < 0.05; Fig. 3B). Moreover, the expression of *ZFX* variant 1/3 decreased with an increasing stage of breast tumors (*p*< 0.05; Table 2). Additionally, *ZFX* variant 1/3 was significantly overexpressed in the luminal A subtype of breast cancer compared to other types of breast cancers (*p* = 0.02) and in lymphatic invasion-positive samples compared to negative ones (Fig. 4A). A significant association was also observed between the expression of *ZFX* variant 1/3 and the clinicopathological properties of tumors such as HER2, P53, ER, and the PR status. Moreover, the expression level of *ZFX* variant 1/3 was higher in HER2-positive and PR-negative tumor samples compared to HER2-negative and PR-positive samples, respectively (*p* < 0.05, Table 2). Further analysis showed that there was no variation in the expression level of *ZFX* variant 1/3, according to the P53 and ER status of the tumor samples (Table 2).

### Overexpression of *ZFX* variant 4 in high-grade and ductal breast tumor tissues

We observed that *ZFX* variant 4 was expressed in both cancerous and non-cancerous breast tissues. *ZFX* variant 4 was expressed in 77% (27/35) of the tumor samples and 51% (18/35) of the marginal non-tumoral samples, but there was no difference between its expression in tumoral and non-tumoral tissues (*p* = 0.5; Fig. 3C). Moreover, the expression level of *ZFX* variant 4 was significantly higher in ductal breast tumors in comparison to lobular tumors (*p* < 0.05; Table 2). Furthermore, our results revealed that the *ZFX* variant 4 expression was higher in high-grade breast cancer tissues compared to the low-grade ones (*p* < 0.05; Fig. 3B). Moreover, *ZFX* variant 4 was overexpressed in lymphatic invasion samples. Additionally, the expression level of *ZFX* variant 4 was higher in ER- and PR-negative tumor tissues compared to ER- and PR-positive ones, respectively (*p* < 0.05; Table 2). *ZFX* variant 4 was overexpressed in HER2-positive tumors compared to HER2-negative tumor tissues (*p* < 0.05; Table 2). In addition, our data showed that there was no difference between the expression level of *ZFX* variant 4 and the P53 status, age, and tumor size (Table 2).

### Overexpression of *ZFX* variant 5 in low-grade ductal breast tumor tissues

We found that *ZFX* variant 5 was expressed in 80% (28/35) of the tumor tissues and 69% (24/35) of the marginal non-tumor tissues of the breast. The expression of *ZFX* variant 5 was down-regulated in tumor tissues in comparison to marginal non-tumor samples (Fig. 3D), but *ZFX* variant 5 was expressed more strongly in low-grade tumors compared to high-grade tumors (*p* < 0.01; Fig. 3B). Moreover, this variant was overexpressed in high-stage III A tumor tissues (*p* < 0.05; Table 2). Even though the expression of *ZFX* variant 5 was higher in the triple-negative and luminal A subtypes rather than HER2 type and luminal B subtypes, there is not significant association between *ZFX* variant 5 expression levels and breast cancer subtypes (Fig. 4A). The expression of *ZFX* variant 5 significantly elevated in ER-positive (*p* < 0.05), HER2-positive (*p* < 0.05; Table 2), and P53-negative (*p* < 0.05) tumor tissues in comparison to ER-negative, HER2-negative, and P53-positive, respectively. Furthermore, there was a significant correlation between *ZFX* variant 5 expression and tumor size and necrotic tissue (*p* < 0.05; Table 2).

### Expression of *ZFX* variants during differentiation

To further investigate the role of *ZFX* variants in the differentiation, NCCIT (embryonic carcinoma) cells were treated with *trans* retinoic acid to actuate them for differentiation. To confirm the accuracy of the differentiation, the expression level of *NANOG*, *OCT4*, *Nucleostemin*, and *Nestin* were measured. We found that these stem cell markers were down-regulated during differentiation. The *ZFX* variant 1/3 and *ZFX* variant 4 expression reduced during *trans* retinoic acid differentiation, but the expression of *ZFX* variant 5 did not change during *trans* retinoic acid differentiation (Fig. 4B).

## DISCUSSION

Transcription factors with zinc finger domains are involved in proliferation, differentiation, migration, and cancer development. *ZFX* is specially expressed in embryonic stem cells and is responsible for self-renewal and the maintaining of pluripotency of stem cells [6,25], possibly by regulating the balance between self-renewal and differentiation[7]. Recent studies have indicated that *ZFX* is also expressed in cancer cells and tissues[17,20,22,26-28]. ZFX has also found to contribute to signal transduction, stress response, cell cycle regulation, and the metastasis of cancer cells[22,26,27,29,30]. Many splicing regulatory proteins control alternative splicing in cancer, and their dysregulation plays a key role in tumor development and progression. The dysregulation of alternative splicing leads to large-scale changes in the balanced expression of spliced variants of genes, thus contributing to critical pathways involved in tumor initiation and progression[31]. Several studies have demonstrated the differential expression, localization, and function of the spliced variants of self-renewal genes such as *OCT4*-spliced variants in cancer cells and tissues, which may potentially be relevant to other stem cell transcription factors[32-34].

Five different variants are transcribed from the *ZFX* gene that encodes three isoforms. ZFX isoform I is the largest protein with three complete domains that can be in N-glycosylated or un-glycosylated forms. While the N-glycosylated form of the ZFX isoform I is localized in the nucleus, its un-glycosylated form is found in the cytoplasm, suggesting that post-translational modification may be involved in the regulation of the nuclear import of the ZFX isoform I[35]. ZFX isoform II has a truncated transcriptional activation domain of which at least a portion can recognize the DNA target sequence, resulting in qualitatively different regulatory properties[5,36]. ZFX isoform III, which lacks the C_2_H_2_ zinc finger domain, may not be able to recognize or bind to a DNA sequence. Reports have shown the ZFX expression in both cytoplasm and the nucleus of human glioma cell lines[35], colorectal cancer tissues[37], and tongue squamous cell carcinoma tissues, but only cytoplasmic signal has been detected in renal cell carcinoma tissues[38], and only a nuclear signal has been identified in normal samples of tongue tissues[39]. It is suggested that different ZFX isoforms may have different expressions and functions in cancer cells[9]. Therefore, we investigated the possible expressions of *ZFX*-spliced variants in the breast cancer cell lines and 47 breast cancer samples by using specific primer sets. Consistent with previous studies, we have found that *ZFX* variants are expressed in stem cells (NCCIT) and in cancerous cell lines[7,9]. All three variants were expressed in breast cancer cell lines (Fig. 2).

We further evaluated the expression level of variants in tumor vs. non-tumor marginal tissues of 47 patients with breast cancer at the mRNA level to investigate the possible role of *ZFX*-spliced variants in tumorgenesis. While the expressions of *ZFX* variant 1/3 and *ZFX* variant 4 were elevated in tumor vs. non-tumor breast samples, the expression of *ZFX* variant 5 had reverse result. Despite a high expression of *ZFX* variant 4 in high-grade tumors vs. low-grade ones, the expression of *ZFX* variant 1/3 and *ZFX* variant 5 were down-regulated in high-grade tumors in comparison to low-grade ones. Additionally, *ZFX* variant 4 had the lowest expression of all variants during differentiation. The latter findings suggest that *ZFX*-spliced variants correlate with the state of cellular differentiation and may be used to predict the degree of malignancy of breast tumors. The correlation between tumor stages and *ZFX* variant expressions was assessed and showed that, contrary to the *ZFX* variant 1/3, the highest level of *ZFX* variant 4 and *ZFX* variant 5 expression was observed in stage III breast tumors. High-stage breast cancer is often associated with the spread of cancer to healthy tissues. Therefore, based on our results, the different expression patterns of *ZFX* variants in different stages of breast cancer may be involved in the progression and metastasis of breast cancer cells. Our data also showed that the expression of *ZFX* variant 1/3 significantly increased in the luminal A subtype of breast cancer, whereas the expression of *ZFX* variant 4 was elevated in the triple-negative subtype of breast cancer. Also, the expression of *ZFX* variant 5 increased in both triple-negative and the luminal A subtypes in comparison to other subtypes of breast cancer. The distinctive expression patterns of *ZFX* variants may be useful in discriminating the different types of breast tumors. HER2 regulates the proliferation of breast cells, and the *HER2* gene plays a key role in the development of breast cancer[40] via cell proliferation and survival through AKT and MAPK pathways, which are affected by *ZFX* overexpression[35,41]. Here, our results showed a significant association between the HER2 status and the up-regulation of *ZFX*-spliced variants. In conclusion, our data show that the *ZFX*-spliced variants have a distinctive expression pattern in different types of breast cancer, and the variants may contribute to the tumorigenesis of breast cancer. Hence, *ZFX*-spliced transcripts may be considered as novel tumor markers with potential diagnostic, prognostic, and therapeutic values.

## References

[1] Alison MR, Lin WR, Lim SM, Nicholson LJ (2012). Cancer stem cells:in the line of fire. Cancer treatment reviews.

[2] Clarke MF, Fuller M (2006). Stem cells and cancer:two faces of eve. Cell.

[3] Ivanova NB, Dimos JT, Schaniel C, Hackney JA, Moore KA, Lemischka IR (2002). A stem cell molecular signature. Science.

[4] Fortunel NO, Otu HH, Ng HH, Chen J, Mu X, Chevassut T, Li X, Joseph M, Bailey C, Hatzfeld JA, Hatzfeld A, Usta F, Vega VB, Long PM, Libermann TA, Lim B (2003). Comment on “'Stemness':transcriptional profiling of embryonic and adult stem cells” and “a stem cell molecular signature”. Science.

[5] Schneider-Gadicke A, Beer-Romero P, Brown LG, Mardon G, Luoh SW, Page DC (1989). Putative transcription activator with alternative isoforms encoded by human *ZFX*gene. Nature.

[6] Galan-Caridad JM, Harel S, Arenzana TL, Hou ZE, Doetsch FK, Mirny LA, Reizis B (2007). Zfx controls the self-renewal of embryonic and hematopoietic stem cells. Cell.

[7] Harel S, Tu EY, Weisberg S, Esquilin M, Chambers SM, Liu B, Carson CT, Studer L, Reizis B, Tomishima MJ (2012). ZFX controls the self-renewal of human embryonic stem cells. PloS one.

[8] Rahmati S, Emadi-Baygi M, Nikpour P, Emadi-Andani E (2014). Expression profile of ZFX isoform3/variant 5 in gastric cancer tissues and its association with tumor size. Iranian journal of basic medical sciences.

[9] Nikpour P, Emadi-Baygi M, Mohammad-Hashem F, Maracy MR, Haghjooy-Javanmard S (2012). Differential expression of ZFX gene in gastric cancer. Journal of biosciences.

[10] (2008). World Cancer Report.

[11] Ponti D, Costa A, Zaffaroni N, Pratesi G, Petrangolini G, Coradini D, Pilotti S, Pierotti MA, Daidone MG (2005). Isolation and*in vitro*propagation of tumorigenic breast cancer cells with stem/progenitor cell properties. Cancer research.

[12] Voduc KD, Cheang MC, Tyldesley S, Gelmon K, Nielsen TO, Kennecke H (2010). Breast cancer subtypes and the risk of local and regional relapse. Journal of clinical oncology.

[13] Carey LA, Cheang MCU, Perou CM, Harris JR, Lippman ME, Morrow M, Osborne CK (2014). Genomics, Prognosis, and Therapeutic Interventions.

[14] Afzali A, Emadi-Baygi M, Nikpour P, Nazemroaya F, Kheirollahi M (2015). Expression of ZFX gene correlated with the central features of the neoplastic phenotype in human brain tumors with distinct phenotypes. Advanced biomedical research.

[15] Fang J, Yu Z, Lian M, Ma H, Tai J, Zhang L, Han D (2012). Knockdown of zinc finger protein, X-linked (ZFX) inhibits cell proliferation and induces apoptosis in human laryngeal squamous cell carcinoma. Molecular and cellular biochemistry.

[16] Huang D, Gao Q, Guo L, Zhang C, Jiang W, Li H, Wang J, Han X, Shi Y, Lu SH (2009). Isolation and identification of cancer stem-like cells in esophageal carcinoma cell lines. Stem cells and development.

[17] Lai KP, Chen J, He M, Ching AK, Lau C, Lai PB, To KF, Wong N (2014). Overexpression of ZFX confers self-renewal and chemoresistance properties in hepatocellular carcinoma. International journal of cancer.

[18] Tan Z, Zhang S, Li M, Wu X, Weng H, Ding Q, Cao Y, Bao R, Shu Y, Mu J, Ding Q, Wu W, Yang J, Zhang L, Liu Y (2013). Regulation of cell proliferation and migration in gallbladder cancer by zincfinger X-chromosomal protein. Gene.

[19] Zhou Y, Su Z, Huang Y, Sun T, Chen S, Wu T, Chen G, Xie X, Li B, Du Z (2011). The Zfx gene is expressed in human gliomas and is important in the proliferation and apoptosis of the human malignant glioma cell line U251. Journal of experimental and clinical cancer research.

[20] Yang H, Lu Y, Zheng Y, Yu X, Xia X, He X, Feng W, Xing L, Ling Z (2014). shRNA-mediated silencing of ZFX attenuated the proliferation of breast cancer cells. Cancer chemotherapy and pharmacology.

[21] Li K, Zhu ZC, Liu YJ, Liu JW, Wang HT, Xiong ZQ, Shen X, Hu ZL, Zheng J (2013). ZFX knockdown inhibits growth and migration of non-small cell lung carcinoma cell line H1299. International journal of clinical and experimental pathology.

[22] Wu S, Lao XY, Sun TT, Ren LL, Kong K, Wang JL, Wang YC, Du W, Yu YN, Weng YR, Hong J, Fang JY (2013). Knockdown of ZFX inhibits gastric cancer cell growth*in vitro*and*in vivo*via downregulating the ERK-MAPK pathway. Cancer letters.

[23] Fang Q, Fu WH, Yang J, Li X, Zhou ZS, Chen ZW, Pan JH (2014). Knockdown of ZFX suppresses renal carcinoma cell growth and induces apoptosis. Cancer genetics.

[24] Gasimli L, Stansfield HE, Nairn AV, Liu H, Paluh JL, Yang B, Dordick JS, Moremen KW, Linhardt RJ (2013). Structural remodeling of proteoglycans upon retinoic acid-induced differentiation of NCCIT cells. Glycoconjugate journal.

[25] Fang X, Huang Z, Zhou W, Wu Q, Sloan AE, Ouyang G, RE M, Yu JS, Rich JN, Bao S (2014). The zinc finger transcription factor ZFX is required for maintaining the tumorigenic potential of glioblastoma stem cells. Stem cells.

[26] Yan X, Yan L, Su Z, Zhu Q, Liu S, Jin Z, Wang Y (2014). Zinc-finger protein X-linked is a novel predictor of prognosis in patients with colorectal cancer. International journal of clinical and experimental pathology.

[27] Fang Q, Fu WH, Yang J, Li X, Zhou ZS, Chen ZW, Pan JH (2014). Knockdown of ZFX suppresses renal carcinoma cell growth and induces apoptosis. Cancer genetics.

[28] Sakhinia E, Glennie C, Hoyland JA, Menasce LP, Brady G, Miller C, Radford JA, Byers RJ (2007). Clinical quantitation of diagnostic and predictive gene expression levels in follicular and diffuse large B-cell lymphoma by RT-PCR gene expression profiling. Blood.

[29] Tricoli JV, Bracken RB (1993). ZFY gene expression and retention in human prostate adenocarcinoma. Genes chromosomes cancer.

[30] Li K, Zhu ZC, Liu YJ, Liu JW, Wang HT, Xiong ZQ, Shen X, Hu ZL, Zheng J (2013). ZFX knockdown inhibits growth and migration of non-small cell lung carcinoma cell line H1299. International journal of clinical and experimental pathology.

[31] David CJ, Manley JL (2010). Alternative pre-mRNA splicing regulation in cancer:pathways and programs unhinged. Genes and development.

[32] Asadi MH, Mowla SJ, Fathi F, Aleyasin A, Asadzadeh J, Atlasi Y (2011). OCT4B1, a novel spliced variant of OCT4, is highly expressed in gastric cancer and acts as an antiapoptotic factor. International journal of cancer.

[33] Wezel F, Pearson J, Kirkwood LA, Southgate J (2013). Differential expression of Oct4 variants and pseudogenes in normal urothelium and urothelial cancer. The American journal of pathology.

[34] Bayat H, Fathi F, Peyrovi Ho, Mowla SJ (2013). Evaluating the expression of self-renewal genes in human endothelial progenitor cells. Cell journal.

[35] Zhu Z, Li K, Xu D, Liu Y, Tang H, Xie Q, Liu J (2013). ZFX regulates glioma cell proliferation and survival*in vitro*and*in vivo*. Journal of neurooncology.

[36] L'Haridon M, Paul P, Xerri JG, Dastot H, Dolliger C, Schmid M, de Angelis N, Grollet L, Sigaux F, Degos L, Gazin C (1996). Transcriptional regulation of the MHC class I HLA-A11 promoter by the zinc finger protein ZFX. Nucleic acids research.

[37] Jiang J, Liu LY (2015). Zinc finger protein X-linked is overexpressed in colorectal cancer and is associated with poor prognosis. Oncology letters.

[38] Li C, Li H, Zhang T, Li J, Ma F, Li M, Sui Z, Chang J (2015). ZFX is a strong predictor of poor prognosis in renal Cell carcinoma. Medical science monitor.

[39] Yin J, Jiang Y, Wu H, Wang J, Zhang S, Liu H (2015). Overexpression of ZFX and its involvement in squamous cell carcinoma of the tongue. Oncology reports.

[40] Tai W, Mahato R, Cheng K (2010). The role of HER2 in cancer therapy and targeted drug delivery. Journal of controlled release.

[41] Yan X, Shan Z, Yan L, Zhu Q, Liu L, Xu B, Liu S, Jin Z, Gao Y (2016). High expression of Zinc-finger protein X-linked promotes tumor growth and predicts a poor outcome for stage II/III colorectal cancer patients. Oncotarget.

